# Patient, Carer and Professional Perspectives on Barriers and Facilitators to Quality Care in Advanced Heart Failure

**DOI:** 10.1371/journal.pone.0093288

**Published:** 2014-03-27

**Authors:** Susan Browne, Sara Macdonald, Carl R. May, Una Macleod, Frances S. Mair

**Affiliations:** 1 General Practice and Primary Care, Institute of Health and Wellbeing MVLS, University of Glasgow, Glasgow, United Kingdom; 2 Faculty of Health Sciences, University of Southampton, Southampton, England, United Kingdom; 3 Hull York Medical School, University of Hull, Hull, United Kingdom; University of Stirling, United Kingdom

## Abstract

**Background:**

Those with advanced heart failure (HF) experience high levels of morbidity and mortality, similar to common cancers. However, there remains evidence of inequity of access to palliative care services compared to people with cancer. This study examines patient, carer, and professional perspectives on current management of advanced HF and barriers and facilitators to improved care.

**Methods:**

Qualitative study involving semi-structured interviews and focus groups with advanced HF patients (n = 30), carers (n = 20), and professionals (n = 65). Data analysed using Normalisation Process Theory (NPT) as the underpinning conceptual framework.

**Findings:**

Uncertainty is ubiquitous in accounts from advanced HF patients and their caregivers. This uncertainty relates to understanding of the implications of their diagnosis, appropriate treatments, and when and how to seek effective help. Health professionals agree this is a major problem but feel they lack knowledge, opportunities, or adequate support to improve the situation. Fragmented care with lack of coordination and poor communication makes life difficult. Poor understanding of the condition extends to the wider circle of carers and means that requests for help may not be perceived as legitimate, and those with advanced HF are not prioritised for social and financial supports. Patient and caregiver accounts of emergency care are uniformly poor. Managing polypharmacy and enduring concomitant side effects is a major burden, and the potential for rationalisation exists. This study has potential limitations because it was undertaken within a single geographical location within the United Kingdom.

**Conclusions:**

Little progress is being made to improve care experiences for those with advanced HF. Even in the terminal stages, patients and caregivers are heavily and unnecessarily burdened by health care services that are poorly coordinated and offer fragmented care. There is evidence that these poor experiences could be improved to a large extent by simple organisational rather than complex clinical mechanisms.

## Introduction

Heart failure is a terminal condition with a greater number of expected life-years lost [1] than many common cancers. Although outcomes are improving the median survival following a first episode of heart failure is just 2.34 years in men and 1.79 years in women [2]. Such statistics provide a stark picture of a disease that is both an important public health problem and a devastating disease for many people. Much is known about the unmet needs of those with advanced heart failure [3]–[9]. Those with advanced heart failure experience distressing symptoms, such as pain, anxiety and shortness of breath, that lead to poor quality of life [10]–[13] and the importance of addressing and treating such distressing symptoms has been emphasised [8]. Both patients and caregivers often feel unsupported [4]. Access to palliative services are uneven compared to those available for people with cancer; and prognostication is widely acknowledged as a major challenge [9], [14], [15].

The importance of palliative care for those with advanced heart failure and the need to address end of life issues are now well established [16]–[19]. Prominence has been given to the need to use “knowledge of treatment advances and comfort measures” [20] to improve the care for those with advanced heart failure. Nevertheless, despite the rhetoric, nearly two decades of research and the incorporation of much of this information into guidelines for the management of heart failure [17], [19], [21], [22] recent systematic reviews of the literature [6], [7], [23] show that major challenges to high quality care remain. The provision of palliative care services, though advocated, remains patchy at best [19], [24]. In this study, we sought to understand these challenges and identify what needs to be done to improve care: comparing the perspectives of patients, caregivers, and professionals.

## Methods

### Ethics statement

Ethical approval (reference 10/S0701/20) from West of Scotland REC 3 was obtained for both phases of the study. All participants gave written informed consent before taking part.

### Data Collection

The research was designed in two phases: Phase 1 aimed at patients and caregivers; while Phase 2 was aimed at health professionals. Both employed qualitative research techniques to address the study aims. Normalisation Process Theory (NPT) [25], [26] was used to underpin our interview guides and data interpretation.

The interviews for both phases were carried out by SB, an experienced health services researcher. In both phases interviewing was stopped when interviews revealed no new experiences or insights.

### Phase 1 Sampling, Recruitment and Data Collection

A purposive sampling strategy was used to identify patients with advanced heart failure served by one Health Board in Scotland. Those with advanced heart failure patients were deemed study eligible if they met all of the following criteria:

- Grade 3 or 4 NYHA classification HF;
- Were symptomatic despite optimal therapy;
- Had a history of admissions/multiple health care contacts for this condition.

Exclusion criteria included:

- a history of mental impairment that would suggest that they would be unable to give informed consent to participate in the study;
- inadequate spoken English that would prevent participation in an interview undertaken in English.

Recruitment was via a heart failure liaison service; primary care; a Heart Function and Supportive Care Clinic; and local hospital admission units.

Participants took part in up to two semi-structured interviews lasting between 30–90 minutes. Caregivers had the option of participating in a combined interview with the patient or a one to one interview. Participants were asked to comment on their experiences relating to: their heart condition; the care they had received; and thoughts on what could be done to improve care. We specifically asked patients and caregivers how they made sense of their condition and planned for the future and what part health professionals played in this. We explored who they interacted with on a daily basis to help with their care and what additional help they would have liked as well as what they perceived as the main barriers to provision of high quality care and how these might be overcome. We also asked them to describe the things they had to do to manage their condition. Finally, we asked them to reflect on previous admission experiences, exploring what factors they believed contributed to their admission and readmission rates generally for those like themselves, and their ideas about alternatives to unscheduled admission.

### Phase 2 Sampling, recruitment and data collection

A purposive sampling strategy was used to identify health professionals who encounter advanced heart failure patients. We sought the perspectives of specialists in heart failure and palliative aspects of care, as well as those responsible for care in the community. Health professionals took part in focus groups and individual interviews, in which they reflected on patient and caregiver experiences captured in Phase 1 which were presented to them in the form of clinical vignettes. They were additionally asked to comment on factors that might promote or inhibit optimal care for advanced heart failure patients.

### Data Analysis

Interviews and focus groups were recorded and transcribed verbatim. This qualitative data was then analysed using directed content, or ‘framework’ analysis [27]. We developed a coding framework that linked data categories to an explanatory model provided by Normalisation Process Theory [25], [28]. This enabled us to focus on patients' and caregivers' work of managing a terminal condition. We examined their accounts of ‘coherence’ (sense making work) such as learning about illness and treatments; ‘cognitive participation’ (relationship work), for example, arranging help and support to manage illness and treatments; ‘collective action’ (enacting work) which included the work of taking multiple medications; and ‘reflexive monitoring’ (appraisal work) such as reviewing and altering management plans (Table 1). We have demonstrated that NPT is useful in understanding treatment burden experienced by heart failure patients [28] and the coding frame created during that study was used as the starting point for our analysis of data in the current study. As data was analysed iteratively, this coding frame was expanded and refined to accommodate the data in a sensible way (see expanded coding frame Table 1). We took a robust approach to analysis: all the patient and caregiver data was double coded by two parties independently with comparison of results and discussion to ensure uniformity of coding; we used “data clinics”, where the authors coded a sample of transcripts together, in order to further ensure consistency and validity of findings. For the health professional data we again used a framework approach to data analysis but for this work we specifically mapped the health professional responses against the themes identified in Phase 1, in order to help us characterise health professional responses in relation to the issues raised by patients and their caregivers.

**Table 1:** Normalisation Process Theory Coding Frame for Advanced Heart Failure.

COHERENCE - sense making work	COGNITIVE PARTICIPATION - relationship work	COLLECTIVE ACTION - enacting work	REFLEXIVE MONITORING - appraisal work
**Learning about Illness and Consequences**	**Engaging with Others**	**Methods for Managing Symptoms and Treatments.**	**Monitoring Illness or Treatments.**
**Differentiation:** Developing an understanding of the diagnosis, treatments or care, the role of different health professions in the illness. Describing how a symptom feels, and attributing it to certain disease processes, or to other processes such as ageing or medication side-effects.	**Enrolment:** Engaging with friends, family or health professionals to enable them to provide support or advice, and understanding the emotional distress of others due to one's own illness	**Skill set workability:** Developing methods for coping with therapeutic interventions (including medication regimes) and developing strategies to cope with symptoms, exacerbations or emergency situations.	**Reconfiguration:** Altering treatment regime to fit in with daily activities, including rearranging appointments.
**Communal Specification:** Making sense of the illness, diagnosis, investigations or treatments through interactions with others, including health professionals.	**Activation:** Arranging help (logistical, administrative, or expert) to manage the illness, symptoms and treatments from health professionals, social services or friends and family.	**Contextual integration:** Integrating the illness into social circumstances including: installing adaptations to the home or for mobility, moving house, altering social activities due to illness or its management.	**Communal Appraisal:** Discussing, altering or reviewing management plans, getting advice about symptoms, and deciding whether to seek medical attention in discussion with others (either professionals or friends and family).
**Individual Specification**: Researching the illness and its management through medical resources or other media or otherwise reaching one's own personal view of the illness and its management.	**Initiation:** Utilizing one's own skills to contribute to managing illness including: initiating appointments, investigations or treatments, organizing social care, benefits, or following up test results.	**Interactional workability:** Enacting treatments: for example the work of taking multiple medications or attending appointments and tests related to the provision of the treatment.Enduring symptoms of heart failure or other illness, enduring treatments, or side effects of treatments, enduring incongruent interactions with health professionals, and enduring interventions or intrusions from family and friends.	**Individual Appraisal:** Making one's own decisions about the illness and the cause of medical symptoms, about whether to follow medical advice and treatments, and whether to seek medical attention. Reflecting on care and health status,
**Internalization:** Relating how one feels (including frustration, coping, and emotional work) about the treatments, the illness, its prognosis, and understanding the limitations imposed.	**Legitimation:** Seeking or providing reassurance about treatments from friends, family or professionals or dealing with stigmatization and mismatches in ideas and expectations from others regarding one's illness.	**Relational integration:** Describing relationships with, and confidence in, medical professionals and coping with multiple health professionals as care givers and poor communication between them. Overcoming barriers to gaining access to care.	**Systematization:** Keeping up to date with new information about the illness or new treatments and developing a routine for self monitoring.

### Participants

Table 2 provides details of the 30 advanced heart failure patients included in the study. The 20 close persons consisted of eleven female partners; five male partners; three women who were daughters or a sibling and one son. The age range of those with advanced heart failure was 60–86 years, with 8 females and 22 males. The mean number of prescribed medications was 15 (range 5–27); while the number of comorbidities ranged from 2–9 with a mean of 5. The Scottish Index of Multiple Deprivation (SIMD) was used to measure deprivation (ref http://www.scotland.gov.uk/Topics/Statistics/SIMD), and is divided into quintiles based on the national scores for Scotland. The index combines information from seven domains which carry different weightings involving: current income (28%), employment (28%), health (14%), education (14%), geographic access to services (9%), crime (5%), and housing (2%). While we had representation from across the socioeconomic spectrum most participants came from more deprived backgrounds. Table 3 provides information about the 65 health professionals.

**Table 2:** Advanced Heart Failure Patient Participants.

Patient Characteristics		Patients (n = 30)
		n
**Age at first interview (years)**	60–69	9
	70–79	16
	≥80	5
	Age range	60–86
	Average age	72
**Sex**	Female	8
	Male	22
**Medications (n)**	Less than 10	3
	10 to 20	24
	More than 20	3
**Co-morbidities (n)**	2–4	10
	5–7	17
	8–9	3
**SIMD Quintile**	east Deprived	3
	2	3
	3	2
	4	10
	Most deprived	12

**Table 3:** Health Professional Participants.

General Practice (GPs, Practice Nurses, District Nurses and Practice Managers)	Focus Groups ×3 (n = 29)
Accident and Emergency Consultant	Interview ×1
Medicine for the Elderly Consultant	Interview ×2
Cardiology Consultant	Interview ×1
Palliative Care Consultant	Interview ×1
Cardiology Trainees	Focus Groups ×2 (n = 14)
Ambulance Service	Interview ×1
Heart Failure Liaison Nurse	Interview ×3
Palliative Nurse (Heart Failure Interest)	Interview ×1
Marie Curie Nurse	Interview ×1
District Nurses	Focus Group ×1 (n = 8)
District Nurse (Out of Hours)	Interview ×1
Palliative Care Pharmacist	Interview ×1
Pharmacist (Pharmacy Heart Failure Service)	Interview ×1

## Findings

Our findings related to four key problems: knowledge and understanding deficits; difficulties navigating and accessing health and social care support; general challenges and barriers to optimal care; and problems relating to emergency care. Illustrative quotations are provided. Of particular interest was the extent to which patients and caregivers on the one hand and health professionals on the other, agreed regarding challenges that need addressed and the key barriers and facilitators to improved care.

### Knowledge and understanding deficits

Patient and caregiver accounts revealed that poor knowledge and misunderstanding of the diagnosis and its implications was ubiquitous as the following comment illustrates:

*“I think it seems to me not like cancer where they say you've got five months to live or you've got a year but nobody has said that. I wonder whether that is a good strategy or what, I don't know, but I really like answers but it's because we have always been in control of our lives and now we are not.” Patient 08*

Participant accounts suggest that a lack of candour about the nature of the disease was a feature of the patient and caregiver experience that contributed to poor understanding of the condition and its consequences.

*“Why was I not told that things were getting worse? I didn't expect them to get any better but I thought they would just be stabilised and he said ‘because my thingy is, I don't believe in telling a patient until they need to know and now you need to know”. Patient 10*

Perhaps because of their poor understanding of their diagnosis some patients failed to recognise the deterioration of their condition over time. While some understood that their condition could not be ‘cured’ or ‘reversed’ they expressed the hope that it would not deteriorate. There was little evidence that many patients were aware of the terminal nature of the condition, even in the very latest stages of the illness.

Both patients and caregivers also had a poor understanding of treatments, their side effects and limitations. This was true for both medications and device therapies. For example, it was clear that patients and caregivers had many misconceptions about the functions of devices such as implantable cardiac defibrillators and the implications of deactivation and described some extremely unsatisfactory exchanges with professionals regarding such issues. Health professionals agreed this was a widespread problem.

*‘They have the perception in their head that if its deactivated* (the ICD) *they may suddenly die, that as soon as its deactivated they will then die, it's like turning off the respirator.’ Palliative Nurse*

Health professionals were sympathetic to patents' uncertainty about the meaning of their diagnosis and about treatments and were aware that inadequate time for communication contributed to poor understanding. They described difficulties communicating patients' complex and poor prognosis, for example, they felt that patients' had unrealistic expectations about, and poor understandings of, a heart failure diagnosis and its trajectory, as illustrated by the following comment:

*‘They will say ‘oh. At least I haven't got cancer’. Heart Failure Specialist Nurse 1*

Consequently, conversations about palliative care were more difficult to introduce and were clearly expected to be more challenging and time consuming. They saw these problems as compounded by cognitive impairment, complicated by co-morbidity and made more difficult by the uncertainty of prognostication. Some professionals stated that they had to consider that patients may not want to know everything regarding their prognosis, perhaps hinting at a degree of paternalism or recognition of denial as a way of coping, the latter seeming likely for some of the patients interviewed.

Current service configurations were seen as the most significant barrier to good communication, as lack of time and continuity were viewed as crucial issues. Professionals were very aware that meaningful conversations about the condition and its implications were likely to be difficult and could not satisfactorily be undertaken within the context of a brief single encounter.

*‘ The cardiologists, the system that they are expected to work in, the environment, the time constraints that they have, that is not conducive to having these significant conversations with patients and you can't have that conversation without actually building in some additional time or support.’ Heart Failure Specialist Nurse 3*

Health professionals were united in agreeing that the care of those with advanced heart failure was extremely important, that current care for this patient group was suboptimal and there was a need for improvement. However, no professional group identified themselves as having key responsibility for those with advanced heart failure and hence for ensuring patients really understood their condition or its implications. Health professionals described a range of obstacles, which did not seem easily rectifiable, that served as barriers to them undertaking a key, care manager role. Heart failure specialist nurses were well placed to address poor knowledge and understanding with on-going reinforcement of information but felt overstretched and short of time for this demanding task. Cardiologists felt constrained by pressure of time in busy hospital clinics. Generalists often felt that they would need specialist advice and support to enable them to identify when patients were entering a terminal phase. It was clear that some professionals lacked confidence and others were unwilling to assume the lead role for care in the terminal phases of this condition for the reasons outlined above.

### Difficulties Navigating and Accessing Health and Social Care Support

**Figure 1:**
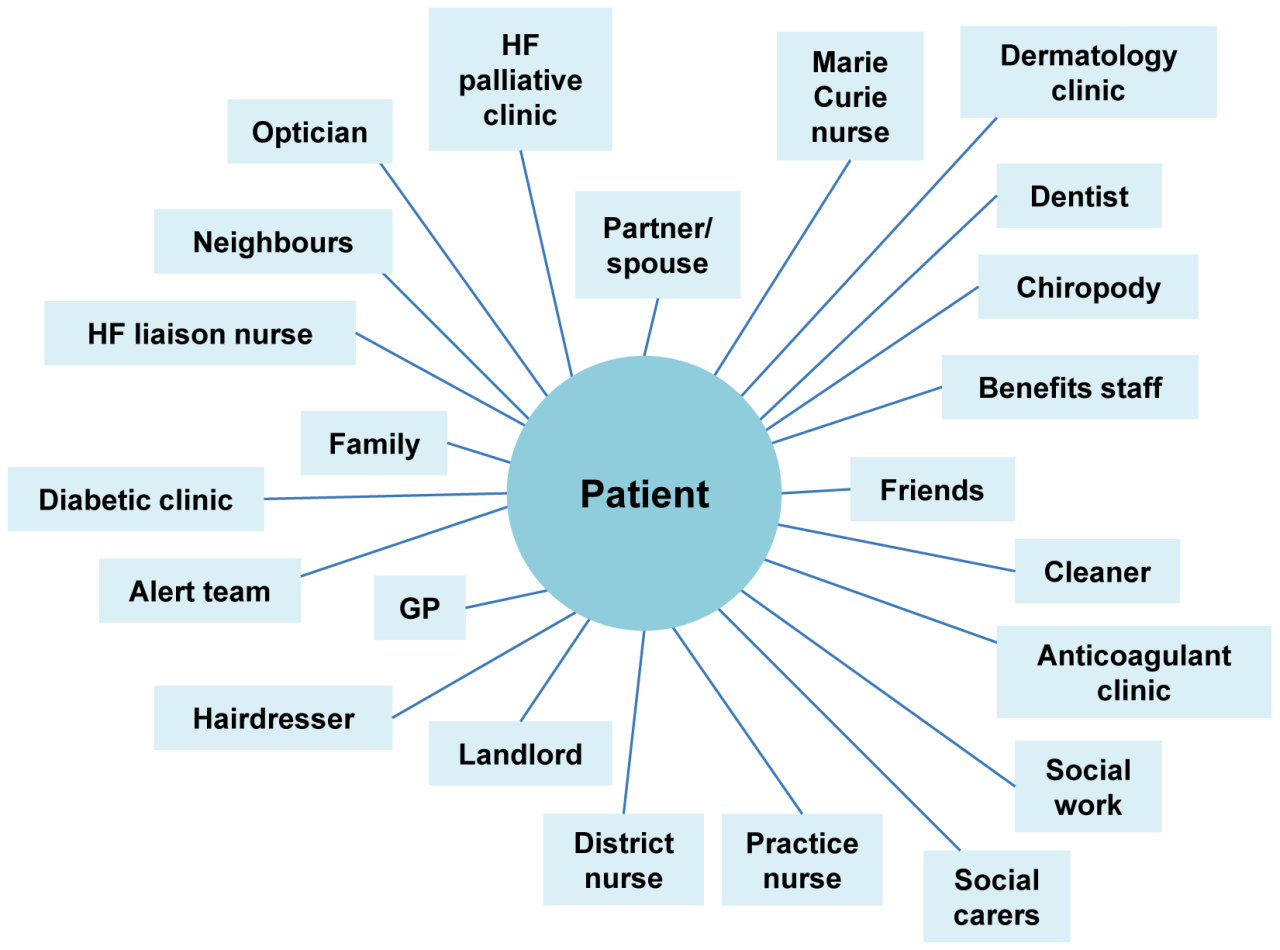
An illustration of the range of people HF patients describe dealing with.

Those with advanced heart failure expended much effort negotiating with a wide range of friends, family and outside agencies to help them with everyday tasks and to access services. Figure 1 illustrates the range of professionals and others people had to deal with and mentioned in their accounts.

Patients sometimes felt that their requests for help were considered illegitimate by others making their situation more difficult. Many different health professionals (primary care physicians and nurses, cardiologists, hospice staff and heart failure liaison nurses) could be involved in providing care, and in the absence of clear care plans, patients and caregivers had to decide who best to contact for usual or emergency care based on their previous experiences of care. Primary care physicians, although generally viewed positively were sometimes perceived as lacking the necessary expertise. A palliative care clinic for heart failure and an outreach heart failure specialist nurse service were generally viewed as useful, often because the nurses helped organise things for patients, but also because both provided continuity and longer appointment times.

*‘And it's the same nurse more or less you get every time you go up. Well she has been to the house, she has spoken to us, she gets to know you. You don't feel you are just a number.’ Patient 11*

Participants described both struggles and delays in obtaining social care support and welfare payments. Health professionals described unequal access to aids and support services for heart failure patients compared to cancer patients.

*‘We probably don't take as good a palliative care approach to them as we should do because they are normally in an emergency medical bed and in a medical ward so they probably don't get the sort of care that they should do if they were say a cancer patient. Because quite definitely I don't think we have that sort of approach palliatively for heart failure patients in hospitals.’ GP Focus Group 1*

Palliative care and hospice services were accessed by only a minority of advanced heart failure patients. This was thought to be related to problems of prognostication and the difficulty identifying the appropriate point to begin palliative care.

*‘Prognostication is kind of entwined in how aggressive you decide to treat them and having an understanding that there comes a point where actually the right thing to do is not to put them back on the IV diuretics but it is to say that you know this is the third time we've been here. This is not going to get better, what is it that you want us to do now?’ Medicine for the Elderly Consultant 1*

Poor levels of patient and caregiver understanding of the disease also made the subject of palliative care difficult for professionals to introduce.

*‘There is resistance because they associate hospices still with death and certainly with the Marie Curie, the Marie Curie name as well, that might be the connection with cancer.’ Palliative Care Pharmacist*

### General challenges and barriers to optimal care

Polypharmacy is a major challenge for patients. Patients invested much time and effort developing routines to help them to remember when and how to take multiple medications in accordance with physician or pharmacist advice, often relying upon caregivers for help and support.

The organisation and delivery of care posed difficulties for patients. They described poorly co-ordinated and disorganised services that did not communicate effectively with each other, and that led to multiple appointments.

*‘X is going to the health centre to see one nurse on a Monday for talking sake, he's having to go back to see another nurse on the Wednesday and then he has got to go back and see somebody else on another day, she says he is down at the same department three times in a week and he could be done in one day. Each of them that, the Sister, the Nurse and the anti-coagulant clinic. She says it's the same building and yet he has got to go three times daily, he's got to go three times a week, different days.’ Close person 12*

Lack of continuity led to lack of consistency in explanation and advice from different health professionals about key aspects of care.

*‘I mean if you are seeing different doctors and they might change something here and then another doctor will say well no we are going to put this one on to that one and your medication is changing a lot.’ Patient 20*

This included advice about what medications were appropriate and whether they might be candidates for specific treatments.

Professionals pointed to the ways that current service configurations acted as a barrier to the delivery of optimal care and failed to promote integrated care. Short appointment times, a lack of nursing and psychosocial support and lack of capacity to provide continuity of care were barriers to the difficult conversations needed to improve patients' understanding of their illness.

*‘You are up against it because the system doesn't work like that, short appointments when people come, don't see the same doctors or nurses, admissions to sort out, you know, the presenting issue, presenting complaint, but not getting to grips with the reasons for repeated admissions. Quick discharges because you need the beds so you are trying to sort out this one area in a system that's actually working against you, so I think that is hugely challenging.’ Palliative Care Consultant*

Communication between health professionals was absent at key points.

*‘There is lack of communication, we just don't quite know what's going on there* (hospital) *and what new services there are, what services have been taken away so it would be very useful to know a bit more.’ GP Focus Group 1*

Hospices were not equipped for active management that many advanced heart failure patients need. A specialist palliative care heart failure clinic model with good links to community medical and social support and long appointment times was seen as the ideal. Advanced heart failure patients were sometimes deemed too complex for generalists to manage and it was suggested that specialist heart failure nurses with an interest in palliative care would be best placed to provide care for this population. There was agreement that the issue of care for advanced heart failure was important but no professional group appeared willing or able to assume responsibility for co-ordinating the complex informational and clinical management of these patients.

*‘Defining roles, as to who does what, like that, like are Marie Curie able to go and stuff with a heart failure patient? And I think there is confusion over all of that.’ District Nurses Focus Group*

*‘I mean I think that the key worker … is absolutely essential in making sure that that care happens and I think that that role is essential in being able to communicate to the key people what's going on.’ Heart Failure Liaison Nurse 3*

### Problems Relating to Emergency Care

Emergency admissions were uniformly described by patients as extremely unsatisfactory.

*‘They have no beds so you are lying down there on a trolley… I've seen me lying down there one night eighteen hours I lay down there and eventually I got put to a ward.’ Patient 30*

Consistently bad experiences of admission processes and in-patient stays meant that, patients resisted seeking help until their situation was desperate.

*‘No the thing is the hospital is the last resort you know what I mean and I wouldn't do it, I wouldn't phone for a doctor or a medic unless I thought there was something seriously wrong.’ Patient 04*

The lack of expert support outside of office hours was unhelpful. Discharge arrangements were also sometimes described as inadequate and could result in further admissions because the problems that had precipitated the initial admission were not satisfactorily resolved.

*‘The nurse came up and tapped me on the shoulder, are you ready to go home? Eight o'clock in the morning. I said I would like to see a doctor before I can get home. No, you are going home. They are desperate for the bed, desperate. So I went down to another wee place and I waited seven hours on them sorting out the medication.’ Patient 01*

Health professionals described unclear pathways leading to patients' unscheduled admissions, often out of hours, via emergency rooms. These were universally deemed to be inappropriate. Patients would benefit from clear information on where to seek appropriate help and from whom, especially outside office hours. In such cases, primary care ‘out of hours services’ tended to advise patients to call for an ambulance to take them to hospital, leading to an admission via the emergency department. Inflexible admission procedures within hospitals and ambulance services, prevented direct access to cardiology and led to patients being admitted to inappropriate wards. Solutions such as advance care planning were seen as having the potential to play a part in preventing unnecessary admissions by facilitating fast tracking of patients to appropriate services including hospice services.

*‘We would be delighted if that happened and you could get direct admissions to these wards (cardiology), you could get the enthusiastic heart failure nurses engaging in the ward instead of having to chase around the place to try and find who is where and a guy in the orthopaedic ward … or the respiratory ward and whatever else it is so we just try and make admissions easier to come about and to arrange, to organise and more pleasant to happen.’ Cardiologist*

*‘I think … if there is that clearer path it keeps everybody right from primary care providers through secondary and through palliative service.’ Cardiology Trainees 2*

## Discussion

### Results in Context

We have demonstrated how patients in this study lacked understanding of their condition and appropriate management. Previous research has also highlighted this as a problem [3], [10], [29], [30]. Poor understanding was pervasive and adversely affected capacity for self-care and decisions about help seeking. Our data illustrate how even in the terminal stages of chronic but lethal illness, patients and carers were heavily and unnecessarily burdened by poorly co-ordinated, fragmented, and discontinuous care. Professionals also described such problems. This resonates with a recent systematic review of the international literature which demonstrated the need to improve care coordination and communication between patients, their families, and health care professionals [7]. Importantly, while health professionals unanimously agreed that a key individual or individuals needed to assume responsibility for overseeing care delivery and coordination, no professional group in this study identified themselves as appropriate candidates for such a role. This is a fundamental issue that needs addressed if we are to make a major difference to care provision for this patient population.

Problems relating to prognostication could prevent palliative care services being offered, so it is clear, that professionals should worry less about this and instead focus on addressing the palliative needs of their patients. This resonates with recent cardiological opinion on this issue [13], [21].

The issues raised here highlight how care for those with advanced heart failure remains suboptimal from a patient and caregiver perspective, and professionals are aware of this. Even though clinical guidelines and health policies have strongly encouraged discussions and planning in end of life care, the literature is clear that poor understanding of the implications of advanced heart failure amongst patients is endemic [13], [31]. The current study highlights that little progress is being made but importantly demonstrates that these problems are to a large extent, structurally induced by the health care systems as they currently operate which are unfit to accommodate the support needs and preferences of those with advanced heart failure. These needs include the opportunity to have multiple conversations taking place over multiple contacts and long appointment times and services configured in ways that facilitate greater continuity. *Integrated care* for those with advanced heart failure requires improved communication mechanisms between health professionals, for example cardiologists and palliative care physicians, and across sectors, for example, across the primary/secondary care interface and health and social care boundaries. Streamlined *admission* pathways that help those with advanced heart failure avoid emergency departments are essential to improve patient and caregiver experiences. *Key workers* need to be identified for advanced heart failure patients, the most appropriate health professional might vary depending on context, but someone needs to be clearly seen to have overall responsibility for patient care. *Such individuals* will need access to additional support and advice from a multidisciplinary team.

### Strengths and Limitations

Our work has a number of strengths and limitations. Our research was limited to a single geographical location within the United Kingdom. Patients in this area had access to a well developed heart failure liaison nurse service, and therefore may be better served than patients in other locations, particularly rural areas where there is less access to such support services. However, our findings resonate strongly with the existing literature in this field [6], [7], [31]. Our work also has a number of important strengths. First, we used a highly regarded theoretical framework to underpin our work. Also by asking health professionals to directly respond to issues identified by patients and caregivers we were able to move beyond the existing descriptive work in this sphere and undertake *explanatory work* to increase understanding of barriers to optimal care and the actions that must be taken for us to improve the experiences of those with advanced heart failure.

Addressing the problems highlighted will not require a further guideline but rather a complete reappraisal of how we deal with chronic but inevitably lethal conditions. Currently, patients and caregivers struggle to navigate complex and fragmented health and social care systems that were not designed to address twenty first century health challenges. Instead, services need to be reconfigured in ways that prioritise patient and caregiver complex care needs [32]. Simply exhorting health professionals to “do better” seems unlikely to make a difference unless at the same time systems and incentives are realigned to facilitate more person centred approaches. At the moment there is evidence that this patient group have poor experiences that could be improved by greater attention to simple organisational rather than complex clinical mechanisms.
